# Safety trial of Floseal® haemostatic agent in head and neck surgery

**DOI:** 10.1308/003588412X13171221590971

**Published:** 2012-07

**Authors:** A Ujam, Z Awad, G Wong, T Tatla, R Farrell

**Affiliations:** North West London Hospitals NHS Trust,UK

**Keywords:** Floseal®, Safety, Surgery, Head and neck

## Abstract

**INTRODUCTION:**

Floseal® (Baxter, Hayward, CA, US) can be of value in reducing blood loss and haematoma rates. The manufacturer’s warnings include allergic reaction, poor wound healing and intravascular thrombosis. We aimed to determine whether Floseal® is safe to use in various head and neck surgery (HNS) procedures.

**METHODS:**

A prospective trial was conducted using Floseal® in 42 various consecutive head and neck surgery procedures. Adverse incidents were used as the main outcome measure, including allergic reaction, wound breakdown, wound infection and thrombosis. Secondary outcome measures included haematoma formation, hospital stay, drain times and output.

**RESULTS:**

No adverse incidents were recorded in the trial period. Two patients developed haematomas and required surgical exploration where a bleeding vessel was identified and dealt with.

**CONCLUSIONS:**

Floseal® is safe to be used in head and neck surgery with no adverse effects. A larger number and a control group are required to ascertain its value in reducing blood loss, haematoma formation, drain usage and hospital stay.

Floseal® (Baxter, Hayward, CA, US) is formed of bovine derived gelatine granules coated in human thrombin. Clinical trials on its use in surgery have been documented over the last decade. Its use in surgery is extensive, ranging from neurosurgery and cardiac surgery through to gynaecological procedures. Much has been published on its effectiveness and safety.^1–4^ In otolaryngology and head and neck surgery (HNS), most of the literature describes its use in managing epistaxis, adenotonsillectomy bleeding and endoscopic sinus surgery.^5–7^ Although generally accepted as both safe and effective, there is evidence to support the association of Floseal® use with unwanted effects such as increased synechia formation after middle turbinate medialisation.^8^ Excessive use or spread of Floseal® into areas not requiring haemostasis can also lead to complications detrimental to the surgical outcome, complications clearly identified by the manufacturer.

Floseal® can be of value in reducing blood loss and haematoma rates and is clinically proven to control bleeding.^3^ It is applied to the surgical site from a syringe as a high viscosity gel that is adherent to wet surfaces. The manufacturer provides exact details of the correct use of the gel and its use should not replace conventional haemostatic measures. There are well documented adverse events relating to the use of Floseal®, including anaemia, atrial fibrillation, infection and bleeding.^3,4^ Furthermore, the manufacturer’s warnings include allergic reaction, poor wound healing and intravascular thrombosis. There are no published data on the use of Floseal® in HNS procedures such as parotidectomy, submandibular gland surgery, thyroidectomy, neck dissection or laryngectomy procedures.

At present, the regular use of haemostatic sealants is not established in otolaryngology HNS, unlike other surgical specialties such as neurosurgery, where in certain centres it is used in around 80% of all cranial and spinal surgery routinely.^9^ The literature states that with HNS procedures such as thyroidectomy, haematoma rates vary from 0.8–2%.^10^ Although relatively low, the consequences can be life threatening, and result from rapid swelling and airway compromise. Any adjunct to current haemostatic control that may improve this complication rate should be actively considered and explored.

With the advancement of surgical techniques in HNS shifting towards minimal access procedures, more traditional open access surgery will become less common. Minimal access procedures such as video assisted thyroidectomy, parathyroidectomy, salivary gland excision and even selective neck dissections will allow for same-day discharge and shorter hospital admissions. The rate of haematoma formation for video assisted thyroid surgery is reported as 0.3%^11^ and an even lower rate may be expected if a haemostatic agent is used.

The prophylactic use of a haemostatic agent in such surgical cases would be a welcomed development should it be demonstrated that lower rates of haematoma formation result, reducing post-operative patient morbidity and improving patient safety. The use of Floseal® in this manner does not substitute for good haemostatic surgical practice during or at the termination of the operation, which continues unchanged. We continue to make use of other traditional (clips, bipolar diathermy, suture ties etc) and newer (Harmonic® scalpel [Ethicon Endo-Surgery, Cincinnati, OH, US] etc) haemostasis methods. The final additional step to traditional insertion of a drain and multilayer closure is for the surgeon to spray Floseal® into the wound as an adjunct to reduce post-operative haemorrhage and haematoma formation.

As a preliminary study in an attempt to explore the potential for regular and routine prophylactic use of haemostatic sealant products at the termination of a major HNS procedure, we aimed to determine whether Floseal® (as one such product) is safe to use in such procedures by recording negative outcomes.

## Methods

A prospective trial was carried out using Floseal® in 42 consecutive HNS procedures in the otolaryngology department of a London hospital. Patients with known sensitivity to bovine products were excluded. There were no ethical concerns as Floseal® was being used routinely in the department at the time of the trial. No sponsorship was sought or received from the manufacturer of Floseal® and the authors confirm there were no other conflicts of interest.

Patient data were collected consecutively over a six-month period. All operations were carried out under general anaesthesia and all were elective cases. As well as noting any history of coagulopathy, all patients had routine clotting tests to identify those at risk of increased bleeding. Only two patients included in this trial were taking blood thinning medication at the time of their procedure. One was on aspirin and another on low molecular weight heparin.

All procedures were undertaken by the authors ZA, TT and RF, who were experienced with the use of the Floseal® product, and its application to the surgical field was in strict adherence to the manufacturer’s guidance. Floseal® was applied at the end of all procedures to the surgical field directly on to the source of bleeding with a syringe and left for two minutes. Any excess Floseal® was irrigated away gently. Any large vessel bleed was dealt with by conventional surgical techniques of suture ties and clips. Floseal® was not applied in close proximity to skin incision sites to avoid skin edge delayed healing as recommended by the manufacturer.

Table 1 outlines the different procedures included in this trial and Table 2 highlights the extent of thyroid surgery performed. Post-operatively, patient length of stay, drain insertion time, bleeding and any complications such as haematoma formation were recorded.

**Table 1:** Procedures included in this trial

Procedure	Number of cases
Thyroid	18	Parotid	7	Submandibular gland	4	Neck lump	5	Neck dissection	3	Laryngectomy	3	Haematoma	2	**Total**	**42**

**Table 2:** The extent of thyroid surgery performed

Procedure	Number of cases
Total thyroidectomy	9
Hemithyroidectomy (initial or completion)	6
Isthmusectomy	3
**Total**	**18**

Adverse incidents were the main outcome measure, including allergic reaction, wound breakdown, wound infection and thrombosis. Secondary outcome measures were haematoma formation, hospital stay, drain times and output.

## Results

A total of 42 procedures were included in the study. The mean patient age was 54 years (range: 13–82 years) and 66% of patients were women (*n*=28). Three patients had relevant past medical history of immunosuppression (diabetes and immunosuppressive medication) and two were current cigarette smokers.

The mean operation time was 111 minutes (range: 30–240 minutes). Skin closure was achieved by sutures in all but one procedure (clips used) with either Prolene® or Monocryl® material (Ethicon, Somerville, NJ, US).

None of our cases developed any adverse incidents to the use of Floseal®, with no patients developing any allergic reaction, wound infection or wound breakdown. Wounds were reviewed in the outpatient clinic two weeks after the procedure to confirm the absence of wound infection.

The mean length of hospital stay was 2.6 nights (range: 0–28 nights), with 10 patients being discharged the same day. With regard to drains, a surgical drain was used in 30 procedures and in 5 cases this was bilateral with the size ranging from 10F to 18F. The average time to drain removal was approximately 60 hours (range: 24–168 hours) with a maximum total drain output of 700ml. Three patients developed complications of haematoma formation post-operatively (Table 3).

**Table 3:** Details of patients with post-operative complications of haematoma formation

	Sex	Age	Procedure	Management	Drain used
Patient A	F	72	Superficial parotidectomy	Return to theatre (after 45 minutes)	No
Patient B	M	66	Parotidectomy	Conservative	Yes (72 hours)
Patient C	F	79	Left hemithyroidectomy	Return to theatre (after 120 minutes)	Yes

Patient A developed a wound haematoma after 45 minutes following a superficial parotidectomy (lasting 60 minutes with no drain inserted initially) and was taken back to theatre where an evacuation procedure was undertaken. A drain was inserted at this procedure with further use of Floseal®. The drain was removed after 48 hours with no complications.

Patient B developed a haematoma following a left parotidectomy (operation time: 100 minutes) that was managed conservatively. The drain inserted during the procedure was removed after 48 hours with no further complications.

Patient C underwent an elective left hemithyroid procedure (lasting 120 minutes) that was complicated by haematoma formation observed while the patient was still in recovery. The patient was taken back to theatre approximately two hours later for evacuation of the haematoma where a small venous bleed was identified and cauterised with bipolar diathermy. Further Floseal® was used and drain insertion was undertaken. The drain was removed after 48 hours with no further complications.

## Discussion

There are many topical haemostatic agents available that use a collagen-based matrix. Floseal® consists of two products (a gelatine matrix and a dehydrated topical thrombin) that are mixed in theatre immediately before use. The final material is excellent at conforming to irregular surgical cavities and clot formation is promoted by the presence of thrombin.

HNS differs from other system surgery. The numerous cranial nerves potentially encountered during surgery are quite unique and nerve dysfunction would be particularly pertinent in the context of vital organ functioning (swallowing, breathing, voice) and facial cosmesis. The complex anatomy of the head and neck region with multiple major vessels as well as the unique presence of larynx/tracheal conduit for air passage therefore makes avoidance of post-operative haemorrhage and haematomas a particular priority for the head and neck surgeon, if rapid airway compromise is to be avoided.

Rapidly evolving post-operative haematomas may within a very short period of time result in irreversible nerve ischaemia and dysfunction, causing vocal cord palsies, facial nerve palsies, airway oedema and obstruction. These complications all have significant morbidity if not potential mortality attached to them. Given the extensive vascular supply to the head and neck region, careful and precise control of bleeding is essential to avoid complications of haematoma formation and catastrophic blood loss.

As a baseline study intended to allow us to plan a more extensive investigation protocol, we have sought to demonstrate that haemostatic sealants such as Floseal have no deleterious effects in regular HNS use. Although available for many years, routine Floseal® use has increased in popularity recently for a variety of general and specialist surgical procedures. It was confined initially to more complex scenarios where intra-operative haemostasis was deemed troublesome but with its usefulness as a surgical aid gradually realised, its potential for a wider prophylactic application and role has started to be debated.

Despite Floseal® being available for over a decade, there are no safety trials that investigate its use in salivary gland surgery, laryngectomy and neck dissections. There are numerous publications on adverse effects of the use of Floseal®, including small bowel obstruction, excessive synechia formation, excessive post-operative pain, foreign body reaction resulting in caseating granulomas and microcalcifications mimicking malignancy.^12–15^ Shashoua *et al* reported a case of Floseal® causing caseating granulomas in the pelvis and abdomen of a patient who had undergone a laparoscopic hysterectomy.^12^ These mimicked metastatic disease but were in fact Floseal® gel causing a foreign body reaction leading to giant cell granulomas. Worryingly, in the head and neck region, a similar finding may result in misdiagnosis and unnecessary further procedures.

In 2009 Thomas and Tawfic reported on three cases of post-operative pelvic pain in patients who had undergone pelvic surgery.^13^ The report describes how a possible allergic eosinophil rich inflammatory response may have resulted in excessive formation of granulation tissue and fibrosis. Although none of our patients reported excessive post-operative pain, this could be an important outcome to measure in any future trials. Indeed, studies on long-term effects of Floseal® in endoscopic sinus surgery demonstrated some evidence that Floseal® causes excessive scar tissue formation and adhesions that require intervention by lysis.^16^ It is conceivable that similar effects may be seen in other head and neck procedures; these may also be associated with both negative functional and pain outcomes.

The primary objective of this study was to evaluate the safety of Floseal® haemostatic agent in HNS. Although there are several published studies on the use of Floseal® for adenoid, nasal and thyroid surgery, no trial has investigated other procedures such as parotidectomy, submandibular gland surgery or neck dissections. In particular, this study aimed to evaluate both the incidence of adverse events and outcome measures. The results of the study confirmed that within a range of HNS procedures, Floseal® is a safe haemostatic material to use for control of bleeding. In this trial only three cases (6%) were associated with haematoma formation and only two required further surgery.

## Conclusions

Our findings provide evidence for the safe, routine use of Floseal® in common HNS procedures, as has been suggested by previous studies in other surgical disciplines, although its use must follow the directions provided by the manufacturer. We plan a multicentre, prospective, randomised controlled trial recruiting larger numbers of patients undergoing HNS to further demonstrate, with statistical significance and exclusion of trial biases, the safety and prophylactic efficacy in routine use of topical haemostatic agents such as Floseal®.
